# Abundance and Diversity of Aerobic Anoxygenic Phototrophic Bacteria in Polar Plant Microbiomes

**DOI:** 10.1111/ppl.70441

**Published:** 2025-08-06

**Authors:** Emilia A. Mäkinen, Ole Franz, Janne A. Ihalainen, Marjo Helander, Riitta Nissinen, Suni A. Mathew, Irma Saloniemi, Kari Saikkonen

**Affiliations:** ^1^ Biodiversity Unit University of Turku Turku Finland; ^2^ The Norwegian College of Fishery Science, UiT The Arctic University of Norway Tromsø Norway; ^3^ Nanoscience Center and Department of Biological and Environmental Science University of Jyväskylä Jyväskylä Finland; ^4^ Department of Biology University of Turku Turku Finland

**Keywords:** aerobic anoxygenic phototrophic bacteria, Antarctic, Arctic, biogeographic patterns, plant microbiota, plant‐microbe interactions

## Abstract

Here, we examined the occurrence of plant‐associated aerobic anoxygenic phototrophic bacteria (AAPB) across polar regions. Recently found in polar soils and cold‐climate plants, AAPBs are photoheterotrophs that rely on environmental organic carbon but capture solar energy via anoxygenic photosynthesis. We revealed the abundance of AAPBs by extracting bacteria from plant tissues and imaging the colonies with bacteriochlorophyll‐based near‐infrared fluorescence. The taxonomic distribution of AAPBs was determined via 16S rRNA gene analysis. From the northern hemisphere, we describe AAPBs from the leaf endo‐ and phyllospheres of numerous sub‐ and Arctic plant species in Northern Finland, Svalbard, and Greenland. In the southern hemisphere, we focused on AAPBs in the root and leaf endospheres and the phyllospheres of *Deschampsia antarctica* in Chilean Patagonia and maritime Antarctica. Additionally, we describe AAPB from the tissues of several other plant species in Patagonia. We found AAPBs commonly associated with the sampled plant species across both hemispheres. A diversity of Alphaproteobacteria was found to contain the AAP capability: at all sampling sites, *Sphingomonas* was the most abundant taxon (up to 60%), while Methylobacteria made up a notable proportion of sub‐Arctic and sub‐Antarctic AAPB samples (up to 32%). In contrast to previous studies describing Methylobacteria frequently in various plant communities, AAP‐containing Methylobacteria were virtually absent from our high‐latitude sites. With diverse AAPB taxa found ubiquitously across polar regions and plant tissues, our results call attention to the potential ecological interaction between AAPBs and their plant hosts.

## 1 Introduction

Adaptations to the seasonal changes in light availability are vital to photosynthetic organisms, particularly in polar regions defined by extremes. At high latitudes, light availability ranges from polar day, a period of continuous daylight during summer, to polar night, a period of continuous darkness during winter (Nelson 2009). Because the latitudinal gradient of the light environment is driven by Earth's axis tilt and the ellipticity of the solar orbit, photoperiodic cues provide a stable and accurate prediction of seasonal change compared to temperature and many other environmental factors (Nelson 2009; Saikkonen et al. 2012, 2024). As such, differences among species in their photoperiodic adaptations and responses to light may mirror their latitudinal distribution ranges (Nelson 2009; Saikkonen et al. 2012, 2024).

Here, we focus on photoactive microbes associated with polar terrestrial plants. The effect of seasonal light availability on plant‐microbe interactions has remained largely ignored, despite the evolution of terrestrial plants having centrally involved coevolving microbes. However, numerous marine and terrestrial bacteria are known to be photosynthetic, phototactic, or light‐sensitive in other ways, and may have a crucial role in, for example, carbon cycling in oceans (Finlay 2002; Rockwell et al. 2006; Smith et al. 2017; Losi and Gärtner 2021). Photoheterotrophic microbes such as proteorhodopsin‐containing bacteria and aerobic anoxygenic phototrophic bacteria (AAPB) are abundant in the aquatic and terrestrial ecosystems of cold climates (Andreote et al. 2009; Cottrell and Kirchman 2009; Atamna‐Ismaeel et al. 2012; Yang and Hu 2022). However, their biogeographic distribution patterns and the factors limiting or driving the occurrence of AAPB taxa remain largely unexplored.

A recent study by Nissinen et al. (2023) demonstrated that AAPBs are common in the cold‐climate plant microbiomes of boreal and oroarctic regions of Fennoscandia. As photosynthetic heterotrophs, these microbes acquire energy from solar radiation but are dependent on organic nutrition from their environment (Yurkov and Beatty 1998; Piwosz et al. 2020). They utilize Bacteriochlorophyll a (BChl *a*) pigments and various organic compounds as electron donors to harvest solar radiation and store energy in the form of ATP (Yurkov and Beatty 1998). Jointly with their diverse physiological and metabolic characteristics, AAPBs have been able to globally inhabit a wide range of habitats and environments challenging to many other organisms (Ritchie and Johnson 2012). Together with mounting results suggesting the ecological and evolutionary importance of plant microbiomes in general, these characteristics confer adaptive advantages to AAPB (Acuña‐Rodríguez et al. 2020; Compant et al. 2010; Hardoim et al. 2015; Cordovez et al. 2019; Trivedi et al. 2020). For example, AAPBs have a more versatile selection of photosensors than similar heterotrophic bacterial strains (Ihalainen et al. 2024). Despite this, the global distribution and ecological importance of AAPB to host plant adaptations are still poorly understood.

The general presence of AAPBs in plant phyllospheres has been reported by metagenomic and cultivation‐based methods, as well as in situ using epifluorescence microscopy (Atamna‐Ismaeel et al. 2012; Stiefel et al. 2013; Remus‐Emsermann et al. 2014; Zervas et al. 2019; Nissinen et al. 2023). The light‐harvesting complex marker gene pufM has been detected abundantly in the phyllosphere metagenomes of soy, rice, clover, and *Arabidopsis* (Atamna‐Ismaeel et al. 2012). Cultivable bacteria from the phyllosphere of winter wheat were specifically searched for AAPB, resulting in AAPB isolates clustering primarily with *Methylobacterium* but also with *Rhizobium*, *Roseomonas*, and *Alsobacter* species (Zervas et al. 2019). AAPBs in the water of tank‐forming bromeliads were detected using the pufM marker (Vergne et al. 2021). It was also previously reported that ~13% of bacteria from clover leaf washes were near‐infrared (NIR) fluorescent, indicating a BChl *a* presence and qualifying for AAPB identification. In the same study, all bacteria isolated based on their NIR‐fluorescence, which also harbored the marker gene, belonged to the genus *Methylobacterium* and produced bacteriochlorophyll in cultivation (Stiefel et al. 2013). However, to our knowledge, the only large‐scale systematic isolation of culturable AAPBs from the phyllo‐ and endospheres of a diversity of plants has been performed by members of our research groups (Nissinen et al. 2023).

Thus far, the environmental factors affecting AAPB biogeography and distribution patterns remain largely unknown. Generally, however, the abiotic stressors characteristic of polar environments often select for convergent functional traits between the Arctic and Antarctic, both in plants and their microbiomes (Pearce et al. 2009; Nissinen et al. 2012; Tojo and Newsham 2012; Poosakkannu et al. 2015; Villaverde et al. 2017; Yang and Hu 2022). In this study, we present the first screening and taxonomic identification of AAPBs in the tissues of high‐latitude plants from both hemispheres. From the northern hemisphere, we provide a characterization of AAPB communities in the endo‐ and phyllospheres of more than 20 Arctic plant species sampled in Greenland, Svalbard, and Northern Finland. From Chilean Patagonia and maritime Antarctica in the southern hemisphere, we describe the assemblage of AAPB in the root and leaf endospheres as well as the phyllospheres of *Deschampsia antarctica* É. Desv, one of the two vascular plant species found native to Antarctica (Convey et al. 2011). We also identify AAPB in the endo‐ and phyllospheres of five additional sub‐Antarctic plant species sampled in Patagonia. Our sampling sites span a broad latitudinal range across both polar regions, from 69.5° to 81.6° N in the Arctic and from 52.1° to 62.1° S in the Southern Hemisphere, capturing a wide spectrum of seasonal variation in daylight. In the Northern Hemisphere, our sites experience extreme shifts between polar day (continuous daylight) and polar night (continuous darkness) as they lie within the Arctic Circle. At northern latitudes in Finland, day length varies from 69 days of polar day in summer to 49 days of polar night in winter. These extremes become more pronounced in Greenland, where sites experience up to 143 days of polar day and 137 days of polar night each year. In contrast, sites in the Southern Hemisphere, which are located outside the Antarctic Circle, do not experience polar day or night, but instead show strong seasonal shifts in day length. In Patagonia, day length varies from 17 h in summer to 8 h in winter, while maritime Antarctic sites exhibit more extreme variation, ranging from 20 h of daylight to just 5 h during the darkest months.

As the biogeography and distribution patterns of plant‐associated AAPB remain unstudied, we aim to answer the following questions: (1) does latitude affect the abundance or the overall richness of AAPB in association with different plant tissues; (2) do AAPB communities or distinct lineages exhibit polar distribution patterns? Generally, the abundance and mobility of bacteria enable reciprocal dispersal between the northern and southern hemispheres (Pearce et al. 2009; Tojo and Newsham 2012; Cox et al. 2016). However, we hypothesize that the climate characteristics and lower diversity of plant hosts in the high‐Arctic and Antarctic (Greenland, Svalbard, and Antarctica) decrease the richness of AAPB relative to lower‐latitude polar regions (Northern Finland and Patagonia). Furthermore, we predict that the assemblage of endospheric AAPB diverges across plant tissues and geographic regions, while airborne transmission enables the prevalence of abundant phyllospheric AAPB at all sites (Pearce et al. 2009; Warren 2022; Nissinen et al. 2023). Due to decreased light availability and the physicochemical limits imposed by plant defenses, we expect BChl *a* production and subsequent AAPB detection to be lower in the root endosphere compared to the above‐ground photosynthetic tissues of plants (Hardoim et al. 2015; Vandenkoornhuyse et al. 2015; Cordovez et al. 2019). Although plant above‐ground surfaces provide a demanding environment for microbial communities (Lindow and Brandl 2003; Vorholt 2012), the mobility of airborne microbes likely supports a high abundance of phyllospheric AAPBs, even at high latitudes. In terms of community assemblage, we hypothesize that the light environment and abiotic characteristics of high latitudes promote the evolution of AAPB communities associated with high latitudes. As the abiotic conditions of polar environments are particularly limiting to plant diversity, they likely drive the evolution of polar plant‐associated AAPB genotypes and plant‐AAPB holobionts adapted to polar climates (Cox et al. 2016; Zhang et al. 2021).

## 2 Materials and Methods

### 2.1 Sampling Sites

In the northern hemisphere, the samples were collected during several sampling campaigns over summer and autumn months in 2014–2022 (Supporting Information S1). The sub‐Arctic sampling sites in Northern Finland mainly represent the oroarctic vegetation zone, dominated by *Empetrum* or *Dryas* heaths. The growing season in these sampling sites is 70–110 days, and the annual mean temperature is around −2°C, with monthly mean temperatures of −12°C in February and 11°C in July. The annual precipitation in the Kilpisjärvi area is 515 mm (Finnish Meteorological Institute, fmi.fi). In Svalbard, the samples were collected from two sites close to Longyearbyen, both representing typical Arctic tundra heath vegetation. The average annual temperature in Longyearbyen is −7°C, with monthly averages ranging from −16°C (March) to 5°C (July). Longyearbyen is located in the polar desert with an annual 180 mm of rain (Data from University Center in Svalbard, unis.no, and Norwegian Meteorological Institute, met.no). In Greenland, the sampling sites were located in the Princess Ingeborg peninsula. The sites represent polar deserts with barren soil, soil crusts dominated by cryptograms, and very low vascular plant coverage and diversity. The climate is dry and cold, with a mean annual temperature of −21°C and monthly average temperatures ranging from −28°C in March to 4°C in July. The mean annual precipitation is 188 mm (Data from the Villum Research Station, villumresearchstation.dk, and Danish Meteorological Institute, dmi.dk).

**FIGURE 1:**
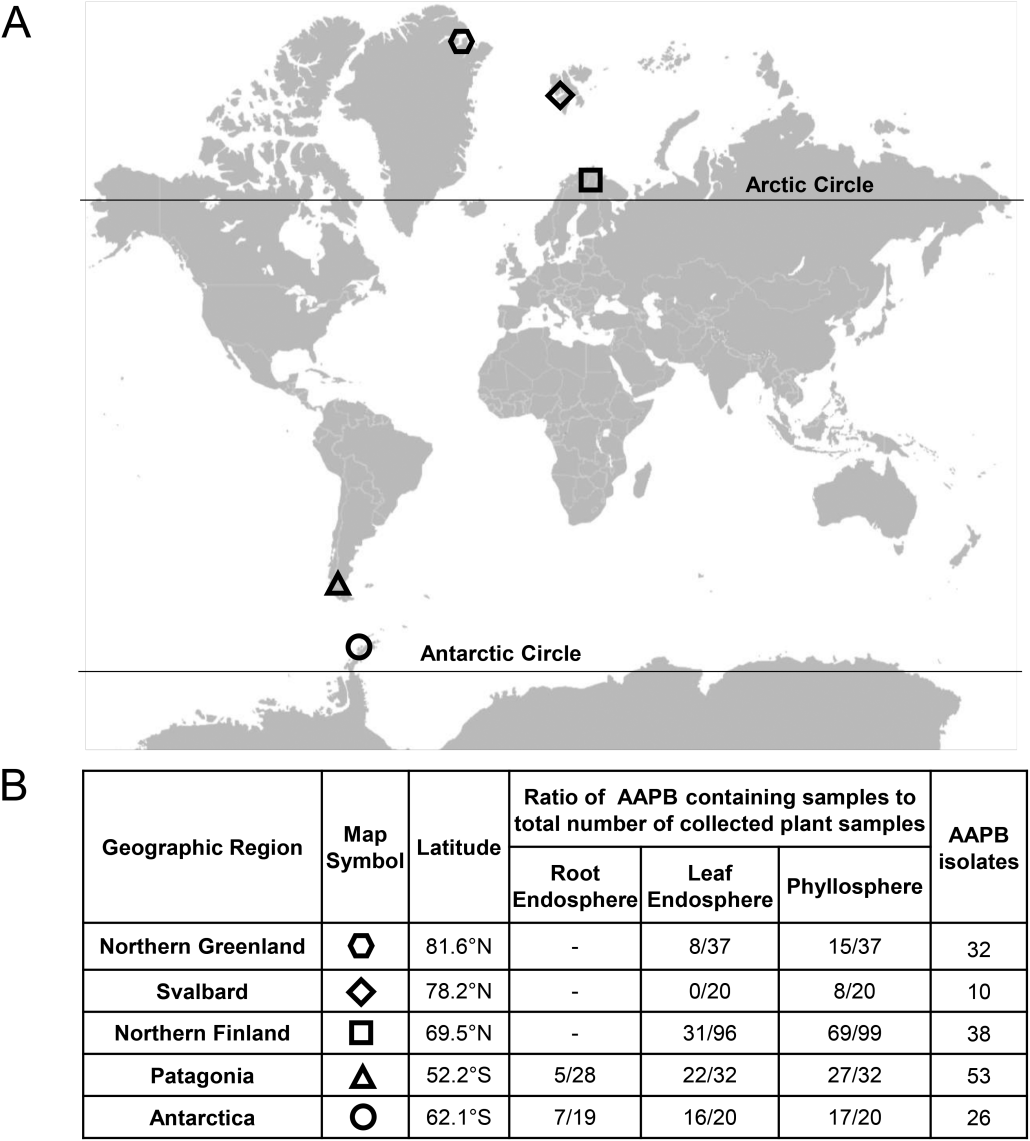
Sampling sites and AAPB occurrence in the collected plant samples. (A) The plant samples were collected from three locations in the northern hemisphere: Northern Greenland, Svalbard, and northern Finland. In the southern hemisphere, the locations were on the Antarctic Peninsula and in Chilean Patagonia. (B) The locations, AAPB occurrence in each part of the plant samples, and the number of collected AAPB isolates (the rightmost column) used for identifying the AAP‐containing microbiome.

In the southern hemisphere, we collected *D. antarctica* plants from 6 Patagonian and 4 Antarctic populations in February 2023 (Supporting Information S1). The examined populations were 50–100 km apart in Patagonia and more than 1 km apart in Antarctica. For the microbe analyses, we used 32 plants from Chilean Patagonia (5–6 plants from each of 6 populations) and 20 plants from King George Island in Antarctica (5 plants from each of 4 populations; Figure 1). The Patagonian populations were growing in dry and windy grasslands with *Empetrum*‐type vegetation. The monthly average temperatures in the area range from 2°C in July to 11°C in January (Data from the Meteorological Direction of Chile, meteochile.gob.cl). The snowy season runs from June until September, with a relatively low average annual precipitation of only 390 mm. The area is also known for its strong winds (gusts up to 100 km h^−1^), which are strongest during the summer. The Antarctic populations were often found close to bird nests, growing among mosses. Monthly average temperatures in King George Island vary from −6°C in July to 1°C during the warmest months (January–February) (Turner et al. 2020). The region receives an annual average precipitation of 700 mm, mostly in the form of snow and drizzle (Pasik et al. 2021).

### 2.2 Sampling, Isolation of Bacteria, and AAPB Identification

For the southern hemisphere samples, plants with their roots and surrounding soil were dug using sterile gloves and garden spades and then placed in sterile plastic bags for transportation in a cooler box. The root and leaf samples were processed in the laboratory within 24 h. In the northern hemisphere, the leaves of the selected plants were harvested with sterilized forceps and scissors into sterile containers and kept in a cooler box until further processing. In the laboratory, plant material was trimmed to remove any dead tissue, while healthy leaves were picked into 15 mL test tubes using sterile tools and subsequently weighed. The time between sample collection and processing never exceeded 72 h for any of the samples and was usually within 24 h. More detailed information on the plant sampling is listed in Supporting Information S1.

Epiphytes were isolated by ultrasonication in sterile 20 mM potassium phosphate buffer, pH 6.5 (KPi), with 0.015% Silwet‐L77 surfactant for 3 min. The extracted microbes were pelleted by centrifugation (13,000 × *g*, 3 min) of 2 × 1 mL of the post‐sonication supernatant. Both pellets were combined and resuspended in a total of 100 μL KPi buffer. For the isolation of endophytes, plant material was surface‐sterilized by a 3‐min immersion in 3% NaOCl, followed by triple rinsing in sterile distilled water (3 × 1 min) (Nissinen et al. 2012). The sterility of the surface was tested by plating 100 μL of the last rinsing water on half‐strength R2A plates. Surface‐sterilized material was homogenized in sterile stomacher bags in KPi buffer using a hammer (5 mL buffer per 1 g of plant tissue).

The macerate (for endophytes) and the resuspended post‐sonication pellet (for epiphytes) were used for serial dilution plating on a half‐strength R2A (Difco) solid medium with the pH adjusted to 6.5. The agar intended for epiphytes contained 50 mg L^−1^ Nystatin as a fungicide. The 50% strength R2A medium has been shown to isolate a wide diversity of endophytes, including Methylobacterial, Acidobacterial, and Bradyrhizobial strains (Nissinen et al. 2012, 2023). The plates were incubated for 3 days at 23°C in ambient indoor lighting, followed by incubation at 4°C in darkness (Hauruseu and Koblížek 2012), and screened for BChl *a‐*containing colonies 4 and 6 weeks after plating. The identification of BChl *a*‐containing colonies allows for a direct screening of functionally active AAPB associated with plant tissues. The screening was performed with a self‐built near‐infrared imaging system (NIRis) which relies on UV‐induced NIR fluorescence of BChl incorporated in the photosynthetic complex of AAPB. Details on the operation of NIRis can be found in (Franz et al. 2024). Total colonies, as well as identified AAPB, were counted for each Petri dish. After the identification, representative AAPB colonies were transferred to new plates and re‐analyzed for BChl α after 10 days (3 days at 23°C, 1 week at 4°C). Individual colonies were streaked repeatedly until pure cultures were obtained, which were preserved in R2B, pH 6.5, with 30% glycerol at −80°C.

### 2.3 Sequencing and Sequence Analysis

Isolates were assigned to bacterial taxa by 16S rRNA gene‐targeted sequencing. The near full‐length gene was amplified using 27F and 1492R primers as described in Nissinen et al. (2012) using freeze‐thawed cell lysates as a template, followed by Sanger sequencing with sequencing primer 1492R. Sequencing was performed by Eurofins Genomics. In addition to bacterial strains isolated and sequenced in this study, 16S rRNA gene sequence data of AAP‐positive *Sphingomonas* strains from previous studies (Nissinen et al. 2012, 2023) were used in the analyses. The partial 16S rRNA sequences were aligned against SILVA SSU NR reference sequence database 138.1, and classified with the least common ancestor (LCA) method at www.arb‐silva.de (Quast et al. 2013). Following established procedure, the sequences were aligned at the genus level as taxonomic classification with 16S rRNA gene sequences becomes unreliable beyond this (Gao et al. 2017). For a full list of classified sequences, see Supporting Information S2.

For phylogenetic analysis of Sphingomonas strains in this study, the partial 16S rRNA gene sequences (801 nucleotides) of Sphingomonas sp. and their closest neighbors from the NCBI nucleotide database were aligned by CLUSTAL W, and the phylogeny was inferred using the Neighbor‐Joining method and evolutionary distances estimated with the Kimura 2‐parameter method (Kimura 1980). The branch support was estimated using bootstrapping (1000 replicates). All phylogenetic analyses were conducted in MEGA 11 (Tamura et al. 2021), and visualized using iTol (Letunic and Bork 2024).

### 2.4 Statistical Analysis

We conducted all statistics in R ver 4.3.2 (R Core Team 2024), with results plotted using packages ‘ggplot2’, and ‘ggpubr’ (Wickham 2016; Kassambara 2023). To qualitatively explore differences in AAPB presence, we performed a binomial generalized linear mixed model on a logit link across all geographic regions combined. Applying procedure glmer from package ‘lme4’ (Bates et al. 2015), we used region (Antarctica, Patagonia, northern Finland, Greenland, or Svalbard), plant tissue (root endosphere, leaf endosphere, and phyllosphere), and interaction as fixed effects, with the sampling location as a random factor. We also considered an alternative model with numerical latitude as the predictor instead of region, and the results remained consistent. To assess the fit of the selected model, we ran dispersion tests and interpreted simulated residual plots with the package ‘DHARMa’ (Hartig 2022). We tested the response variables of the model using type II Wald's chi‐square tests with the package ‘car’ (Fox and Weisberg 2019). To further compare differences in the proportions of AAPB‐containing samples to samples without AAPB, we implemented pairwise contrast tests. Applying procedure glht from package ‘multcomp’ (Hothorn et al. 2008), we performed the contrasts separately for each sampled plant tissue (root endosphere, leaf endosphere, and phyllosphere) to compare differences in AAPB presence between the geographic regions. To control the family‐wise error rate from multiple comparisons, we used Bonferroni‐corrected *p* values adjusted within each tissue.

More detailed analysis of AAP‐positive colonies relative to total culturable communities is limited by the process of selecting representative dilutions from the serial dilution plating and the lack of colony‐count data from the Arctic samples. As such, the significance of comparing the proportions of AAP‐positive to AAP‐negative colonies remains uncertain. However, we performed explorative colony analysis on the *D. antarctica* data from Patagonia and Antarctica to investigate differences in the strength of the AAP signal. For further details, see Supporting Information S3.

## 3 Results

### 3.1 Occurrence of Plant‐Associated AAPB in Polar Regions

AAPB were detected in all sampled plant tissue types and throughout all sampling locations. In our presence/absence approach, AAPB occurrence is indicated by a positive NIR‐fluorescence signal on any Petri dish of a particular plant sample (Franz et al. 2024), but the varying bacterial colony counts were not considered. This approach revealed that the relative occurrence of AAPB diverged across geographic regions (*χ* ^2^ = 41.201, df = 4, *p* < 0.001): the highest proportions of AAPB‐containing plant samples were found in Antarctica and Patagonia, at 68% (*n* = 59) and 59% (*n* = 92), respectively. In contrast, the lowest AAPB presence was observed in Svalbardian plants, where only 20% (*n* = 40) of samples were AAPB‐containing. Although we found no significant interaction in AAPB occurrence between the geographical region and sampled plant tissue (*χ* ^2^ = 3.839, df = 5, *p* = 0.572), the plant tissues (root endosphere, leaf endosphere, and phyllosphere) diverged in their AAPB presence (*χ* ^2^ = 58.901, df = 2, *p* < 0.001). Across all locations, AAPB were found most frequently in the plant phyllospheres, with 65% (*n* = 208) of plant samples being AAPB‐containing. AAPB were least abundant in the root endospheres (20%, *n* = 47), which were only sampled from the Antarctic and Patagonian plants. In comparison, the leaf endospheres harbored a slightly higher presence of AAPB, with 38% (*n* = 205) of plant samples being AAPB‐containing.

**FIGURE 2:**
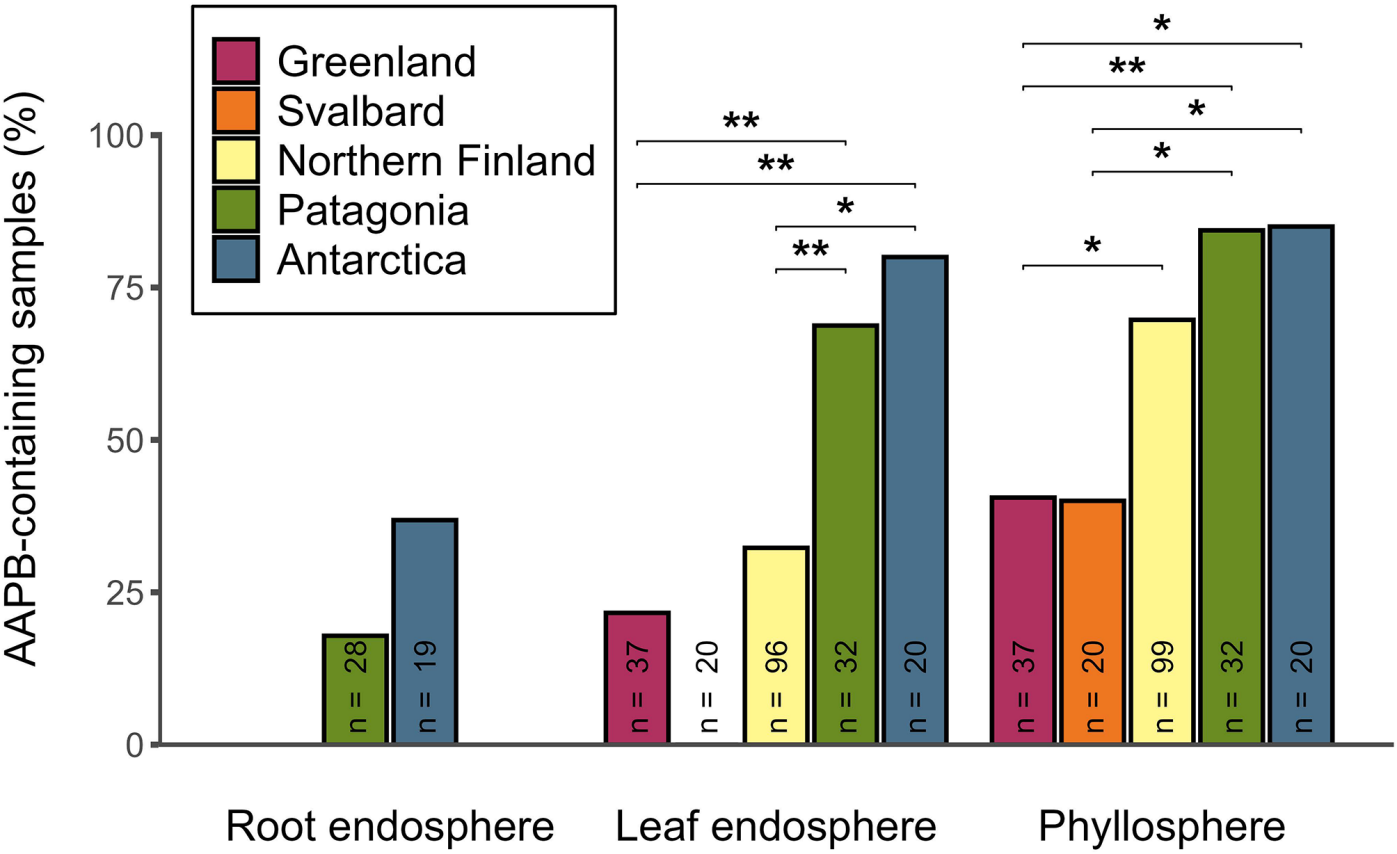
The proportion of AAPB‐containing plant samples from different plant tissues (root endosphere, leaf endosphere, and phyllosphere) between geographic regions (Greenland, Svalbard, northern Finland, Patagonia, and Antarctica). Differences in AAPB presence within the sampled plant tissues across the geographic regions were analyzed via contrast tests. Brackets illustrate significant (*p ≤ 0.05, **p ≤ 0.01) contrasts within each tissue. *n* = number of plant samples.

In all tissue types, samples from the northern hemisphere exhibited generally lower AAPB presence compared to the southern hemisphere. Of the Arctic plants, the proportion of AAPB‐containing samples was highest in northern Finland, both in the leaf endospheres (32%, *n* = 96) and phyllospheres (70%, *n* = 99) (Figure 2). In the phyllospheres, samples from Greenland and Svalbard had similar proportions of AAPB presence, at 41% (*n* = 37) and 40% (*n* = 20), respectively. On the other hand, plants from Svalbard exhibited no AAP signal in the leaf endospheres, whereas Greenlandic samples were moderately AAPB‐containing at 22% (*n* = 37). In the southern hemisphere, the highest proportions of AAPB presence were found in the leaf endo‐ and phyllospheres. Of the leaf endosphere samples, AAPB were found in 69% (*n* = 32) of Patagonian plants and 80% (*n* = 20) of Antarctic plants. In the phyllospheres, the proportions were similar for both origins: 84% (*n* = 32) in Patagonian plants and 85% (*n* = 20) in Antarctic plants. In contrast to the high AAPB presence in the leaf tissues, root endosphere samples were less frequently AAPB‐containing, at 18% (*n* = 28) of Patagonian plants and 37% (*n* = 19) of Antarctic plants (Figure 2).

Differences in AAPB presence were supported by contrast tests between the geographic regions, which showed the sites in the southern hemisphere (Patagonia and Antarctica) to generally diverge from the northern hemisphere (Greenland, Svalbard, and northern Finland), but not between each other (Figure 2). In the leaf endosphere, both Patagonian and Antarctic plants have a higher proportion of AAPB‐containing samples compared to northern Finnish (*t* = 15.210, *p* = 0.005; *t* = 80.840, *p* = 0.020) and Greenlandic plants (*t* = 12.400, *p* = 0.002; *t* = 84.200, *p* = 0.006). However, all pairwise comparisons of AAPB presence between the Arctic regions in the leaf endospheres remained non‐significant. Although Svalbardian samples exhibited no AAPB presence in the leaf endospheres, all contrasts with other regions remained non‐significant due to a large error margin. Contrary to the results on the leaf endospheres, Patagonian phyllosphere samples had a higher proportion of AAPB‐containing samples compared to Svalbard (*t* = 81.350, *p* = 0.017) and Greenland (*t* = 14.980, *p* = 0.005). Similarly, Antarctic samples had a higher AAPB presence in contrast to Svalbard (*t* = 79.070, *p* = 0.037) and Greenland (*t* = 79.710, *p* = 0.030). Furthermore, northern Finnish plants tended to differ from Greenland (*t* = 80.400, *p* = 0.024) in their phyllospheric AAPB presence.

### 3.2 Taxonomic Distribution of Culturable Plant‐Associated AAPB

Based on partial (> 800 nt) 16S rRNA gene sequences, the vast majority of culturable AAPB (*n* = 130 sequenced AAPB isolates) in this study represent Alphaproteobacteria, with only four isolates belonging to Gammaproteobacteria. The major taxa are *Sphingomonas*, unclassified *Sphingomonadaceae* (closely related to the newly described genus *Chioneia*), *Methylobacterium*, and unclassified *Beijerinckiaceae*.

**FIGURE 3:**
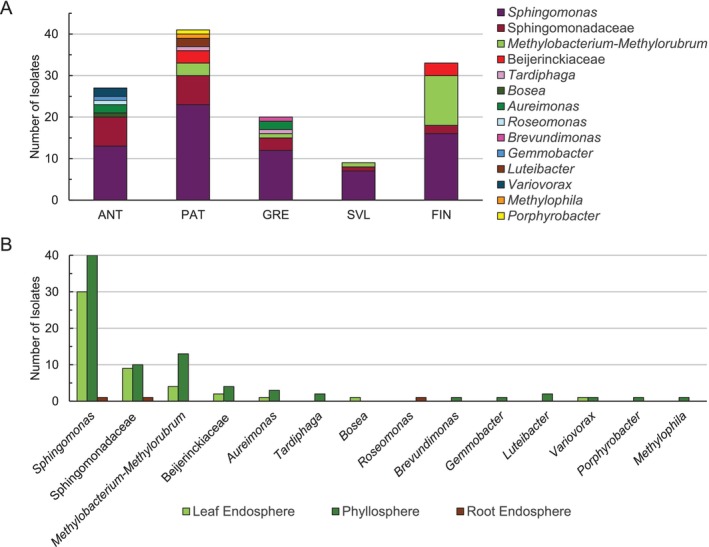
(A) Taxonomic distribution within AAPB communities in different sampling locations. (B) AAPB bacterial genera according to the niche. Taxonomy for the isolates was assigned using the Silva classifier database vs 138.1 (Quast et al. 2013).

*Sphingomonas* was the most abundant taxon in all locations sampled (Figure 3; Supporting Information S2). On average, 56% of all sequenced isolates are classified as *Sphingomonas*. Unclassified *Sphingomonadaceae* were relatively less abundant in the sub‐Arctic sampling locations. In contrast, *Methylobacterium‐Methylorubrum* AAPBs were detected mainly in the sub‐Arctic (Northern Finland) and sub‐Antarctic (Patagonia) regions and were completely absent in samples from Antarctica (Figure 3). Similarly, the unclassified *Beijerickiaceae* strains were only detected in Finnish and Patagonian samples, but not in Antarctic, Greenlandic, or Svalbardian samples. No clear differences were detectable in the taxonomic distribution between the two polar regions. Further, we had a slightly larger number of isolates (77) from phyllosphere samples than from the endosphere (51). Still, essentially all bacterial taxa that could be found more than 10 times were detected both in the phyllospheres and endospheres (Figure 3B), although Methylobacteria isolates were more abundant in the phyllospheres.

**FIGURE 4:**
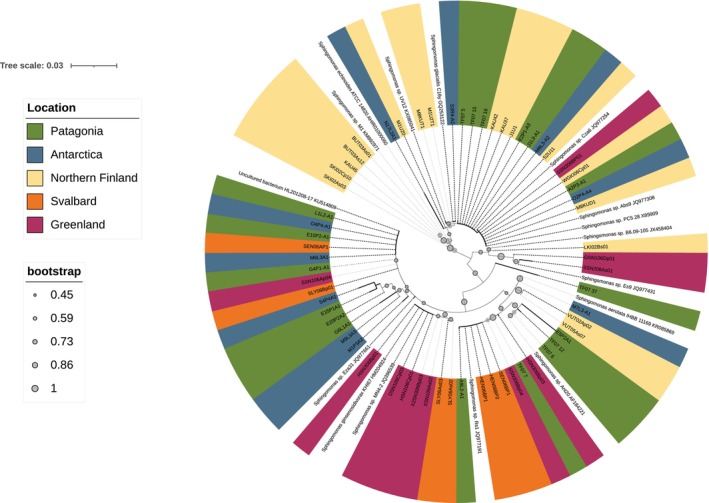
Phylogenetic tree of *Sphingomonas* strains based on the alignment of 16S rRNA sequences from AAPB isolates of this study and most similar sequences from databases (AAP status unknown; uncolored). The evolutionary history represented by the tree was inferred by using the Neighbor‐joining method and the Kimura 2‐parameter model (Kimura 1980). The tree is drawn to scale, with branch lengths measured in the number of substitutions per site. This analysis involved 83 nucleotide sequences; 801 positions of the 16S rRNA gene sequence were used in the final dataset. Evolutionary analyses were conducted in MEGA version 11 (Tamura et al. 2021) and visualized using iToL (Letunic and Bork 2024).

As the *Sphingomonas* isolates represent over 50% of AAPB isolates in this study, we separately analyzed our dataset to decipher possible below‐genus level divergence between the two poles, geographic regions, or niches. The phylogenetic analysis (Figure 4) shows that the major groups of *Sphingomonas* strains cluster with representatives from bacterial species *S. glacialis* , *S. aerolata* , or *S. ginsenosidivorax*. A group of strains from Svalbard and Greenland, most closely related to *S. ginsenosidivorax*, formed a distinct cluster, but in general, we detected no clustering of our *Sphingomonas* AAPB 16S rRNA gene sequences based on sampling location, hemisphere, or niche. The closest neighbors of our *Sphingomonas* strains in sequence databases originate mainly from Antarctica, the Arctic, or the high alpine tundra in the Chinese TianShan mountains, and these were included in the phylogenetic analysis (Figure 4).

## 4 Discussion

Our findings reveal that AAPBs are frequently associated with numerous high‐latitude plant species over both hemispheres. Across the studied polar regions and plant species, we identified a clear AAPB prevalence in both plant endospheric tissues and phyllospheres. In the northern hemisphere, AAPB were most common in the phyllosphere, with slightly less frequent occurrence in leaf endospheres (Figure 2). Furthermore, a geographic divergence was observed in AAPB presence within the northern hemisphere: especially phyllospheric AAPB were more prevalent at the sub‐Arctic sites (Northern Finland), occurring less frequently at the high‐Arctic sites (Greenland and Svalbard). In contrast, we found plants frequently associated with AAPB across all sampled tissue types in the southern hemisphere, with no geographic divergence between Chilean Patagonia and maritime Antarctica. Overall, AAPB were more prevalent in the southern hemisphere than the Arctic, with the difference in AAPB abundance between the leaf endo‐ and phyllospheres remaining less pronounced than in the northern hemisphere (Figure 2). Though only sampled in the southern hemisphere, we found the root endospheres to also exhibit a presence of AAPB. Together, our findings are consistent with a previous study conducted on the two native Antarctic plants, *D. antarctica* and *Colobanthus quitensis* (Kunth) Bartl., which found the total amount of cultivable bacteria to be about one order of magnitude higher in the phyllo‐ than endosphere samples (Araya et al. 2020). Generally, the habitability of plant hosts as ecosystems for either horizontally or vertically transmitted microbes is affected by the environment, plant responses, and ambient conditions within plant structures (Vellend 2010; Hardoim et al. 2015; Vandenkoornhuyse et al. 2015; Cordovez et al. 2019). As such, the prevalence of specific plant‐microbe interactions will likely vary not only on the geographical scale and between plant species but also across different plant tissues. The mobility of airborne microbes and the physicochemical limitations imposed by plant defenses may thus result in a higher diversity and availability of plant‐associated AAPB in leaf phyllospheres compared to internal and below‐ground endospheric tissues (Pearce et al. 2009; Vandenkoornhuyse et al. 2015; Cordovez et al. 2019). However, variation in phyllospheric AAPB occurrence may also be explained by variation in the timing of sample collection. Seasonality likely has a particularly significant effect on AAPB assemblage in the phyllosphere, where ambient conditions (temperature, nutrient availability, humidity, exposure to irradiation, etc.) are more temporally variable compared to the endosphere (Lindow and Brandl 2003; Vorholt 2012). Still, our characterization provides an extensive approximation of the polar AAPB taxa in different plant tissues, as it is well established in literature that up to 80% of plant‐associated bacteria can be cultivated (Nissinen et al. 2012; Bai et al. 2015).

Previous studies investigating AAPB taxa in plant endo‐ and phyllospheres are scarce, and completely absent for most polar habitats and their plant species. However, an analysis of a complete microbiome of *D. antarctica* is available: bacteria from the orders Pseudomonadales, followed by Hyphomicrobiales, are the most abundant in its phyllosphere (Cid et al. 2017; Araya et al. 2020). A cultivation‐based study by Zhang et al. (2020) showed that over 50% of bacteria isolated from both the endospheres and phyllospheres of *D. antarctica* belonged to the phylum Pseudomonadota. The second largest phylum was Actinomycetota for the endosphere isolates and Bacteroidota for the phyllosphere. Consistent with the metagenomic studies, Pseudomonadales is the most abundant order in both niches. While *Sphingomonadaceae* could be found in the endo‐ and phyllospheres of *D. antarctica* , no occurrence of *Methylobacteriaceae* among the more abundant taxa was reported (Zhang et al. 2021). Interestingly, *Methylobacteriaceae* has been by far the biggest group of cultivated AAPB in the study of winter wheat phyllosphere communities (Zervas et al. 2019), as well as in the phyllospheres of spruce, pine, bilberry, and lingonberry (Nissinen et al. 2023) at multiple sub‐Arctic locations. Additionally, all 87 AAPB strains isolated by leaf washes of clover belonged to *Methylobacteriaceae* (Stiefel et al. 2013). In our study, the difference in the occurrence of AAP‐containing *Methylobacteriaceae* between the poles and latitudes was distinct. No single *Methylobacteria* AAPB was isolated from Antarctica, and only at low frequencies (single isolate per location) in Svalbard and Greenland (Figure 3). In contrast, *Methylobacteria* were the second largest group in sub‐Arctic samples and also occurred in samples from Patagonia. Across all sampled sites, AAPB isolates identified as *Sphingomonas* made up the largest share of strains. We hypothesize a taxonomic turnover toward the poles via an increasing share of *Sphingomonas* AAPB, while the share of *Methylobacteriaceae* declines. This would explain why the aforementioned studies on plants from temperate climates resulted in the identification of *Methylobacteriaceae* as the major group of plant‐associated AAPB. However, it cannot be concluded that these differences are purely pole‐dependent, as they are likely influenced by the difference in host‐plant diversity within our sample set. To assess the differences in both the ubiquity and species composition of plant‐associated AAPB along the latitudes, more sampling is needed.

Previous studies on AAPB communities have largely focused on aquatic systems, leaving the distribution of terrestrial plant‐associated AAPB interactions mostly unexplored. Together, our results suggest a potential region‐ or host availability‐dependent pattern in AAPB abundance. Although the sampled plant species differ between hemispheres, we found lower abundances of AAPB at the northern sites situated beyond the Arctic Circle, in contrast to the southern sites located close to the Antarctic Circle. These findings support the hypothesis that geographic isolation, extreme temperature climate, and the light environment of high latitudes may limit the dispersal and survival of certain plant‐associated AAPB taxa. In the Antarctic, we found the proportion of AAPB‐containing plant samples consistent with phytoplankton‐associated AAPB recorded in aquatic communities (Piwosz et al. 2020). In contrast, the proportion of AAPB‐containing leaf samples was higher in our southern sites than what has been previously recorded in boreal forests (Nissinen et al. 2023). On the other hand, we found sub‐ and high‐Arctic plants from the northern hemisphere to support similar frequencies of AAPB in both the endo‐ and phyllospheres to what has been previously reported from Finland by Nissinen et al. (2023). However, the informativity of such comparisons is limited by variations in sample size, the sampled plant species, and the timing of sample collection across the polar sites. Despite these discrepancies, applying the same culture‐based protocol consistently across all samples allows for meaningful comparisons across our geographically diverse dataset. Moreover, our AAPB sampling presents a relatively extensive taxonomic coverage of the limited plant diversity found at high latitudes, as more than 20 woody and herbaceous species were sampled in the northern hemisphere. In the southern hemisphere, the sampled grass species *D. antarctica* represents half of Antarctica's native vascular plant diversity (Convey et al. 2011; Chown et al. 2015), and the five additional species sampled in Patagonia improve our coverage of the region. Though the species specificity and the seasonality of plant‐AAPB interactions remain unknown, some of the observed strains are expected to be associated only with a group of specialized hosts. In both hemispheres, a higher AAPB presence associated with low‐latitude plant tissues is likely related to more suitable climate conditions for microbial growth (Delgado‐Baquerizo et al. 2016; Yang et al. 2021). The milder climate supports an overall higher plant diversity, which may promote higher diversification and abundance of plant‐associated endophytes, as well as a higher survival rate of phyllospheric AAPB (Harrison and Griffin 2020; Yang et al. 2023).

Due to the high dispersal ability of microbes, the observed differences in AAPB occurrence and taxonomic relationships are likely caused by niche constraints imposed by the extreme abiotic conditions of high‐latitude polar regions, including low temperatures and high seasonal variation in light availability (Finlay 2002; Oren 2002; Saikkonen et al. 2012, 2024; Swan et al. 2013; Piwosz et al. 2020). It has been demonstrated that airborne microbes can successfully colonize Antarctica over air currents and extensive bodies of water, promoting the availability of both pathogenic and potentially mutualistic microbe interactions with the plant species present, particularly in phyllospheric tissues (Pearce et al. 2009). As such, differences in AAPB community structures may be shaped by plant‐specific interactions, thus reflecting the distribution history of plant hosts (Hardoim et al. 2015; Cordovez et al. 2019; Harrison and Griffin 2020). Such an effect may be particularly significant in Antarctica, where geographical isolation and limited availability of ice‐free habitat space have functioned as strong filters to plant colonization (Convey et al. 2011; Colesie et al. 2023). Although less extreme, similar factors limit plant diversity in Greenland and Svalbard in the northern hemisphere (Drees and Daniëls 2009; Normand et al. 2013; Birkeland et al. 2017). The availability of a more diverse community of plant hosts at lower latitudes likely supports a more diverse community of plant‐associated microbes (Harrison and Griffin 2020). Therefore, the divergent distribution dynamics of plant hosts between high‐latitude islands and lower polar regions have likely played a role in the evolution of plant‐associated AAPB assemblage and diversity. Though the role of endophytic and phyllospheric AAPB communities in the adaptive functions of their hosts remains virtually unknown (Nissinen et al. 2012, 2023), the availability of plant‐associated microbes able to tolerate the particularly limiting conditions of high latitudes is especially significant to polar plants (Hardoim et al. 2015; Trivedi et al. 2020). As photosensitive bacteria, we suspect AAPB to play a role in the microbial interactions within light‐penetrating tissues of their hosts, particularly the above‐ground external surface and leaf endospheres exposed to ambient light (Compant et al. 2010; Cordovez et al. 2019; Trivedi et al. 2020). The potential effects of these interactions within the host‐specific microbial communities and their significance to host physiology, development, or behavior may become especially relevant at high latitudes, where extreme seasonal variation in light availability is limiting to plant phenology and performance (Alberdi et al. 2002; Nelson 2009; Saikkonen et al. 2012, 2024). To assess the functional role of AAPB in plant dynamics, especially in the biogeographical context of climate change, manipulation experiments together with a wider network of AAPB sampling and isolation of AAPB strains are required.

## Author Contributions

This study was conceptualized by J.A.I., M.H., R.N., and K.S. Sample collection in the northern hemisphere was conducted by O.F., J.A.I., and R.N., whereas in the southern hemisphere by M.H., R.N., and K.S. AAPB were identified by O.F., J.A.I., and R.N., and bacterial sequencing was conducted by O.F., R.N., and S.A.M. Data curation was led by O.F., R.N., and S.A.M., and supported by E.A.M. Statistical analyses were conducted by E.A.M. and supported by I.S. Results were visualized by E.A.M., O.F., and S.A.M. E.A.M. wrote the original draft, which was reviewed and edited by all authors. The composition of details related to AAPB identification, taxonomic analysis, and gene sequencing in the introduction, methodology, and discussion were led by O.F. and R.N., supported by J.A.I.

## Supporting information


**Data S1:** Supporting Information on the plant samples used in this study.
**Tables S1–S3:** Metadata on plant sampling and ratios of AAPB containing plant samples to the total number of samples.

**Data S2:** Supporting Information on AAPB sequences.
**Table S4:** AAPB characterization from three niches (root endosphere, leaf endosphere, and phyllosphere) in the sampled plant species at all sampling locations.

**Data S3:** Supporting Information on an explorative colony analysis of the *Deschampsia antarctica* isolates.
**Figure S1:** Proportion of AAP‐containing to AAP‐non‐containing colonies in different tissues of *D. antarctica* .
**Tables S5 and S6:** Results of statistical comparisons between the AAP signals of different plant tissues and sampling locations.

## Data Availability

Metadata on sample collection and bacterial isolation is available in the Supporting Information of this article. All previously unpublished sequence data in this study have been deposited in the National Center for Biotechnology Information (NCBI) under accession numbers PV697603–PV697726.
