# Supplementary material for: Abundance and Diversity of Aerobic Anoxygenic Phototrophic Bacteria in Polar Plant Microbiomes
Source: Physiol Plant. 2025 Aug 6;177(4):e70441. doi: 10.1111/ppl.70441 (PMC12328840; doi:10.1111/ppl.70441)
Supplement: Supplementary file 3 — Data S3: Supporting Information on an explorative colony analysis of the Deschampsia antarctica isolates. Figure S1: Proportion of AAP‐containing to AAP‐non‐containing colonies in different tissues of D. antarctica . Tables S5 and S6: Results of statistical comparisons between the AAP signals of different plant tissues and sampling locations. [file PPL-177-e70441-s001.pdf]

Supporting information

Abundance and diversity of aerobic anoxygenic phototrophic bacteria in polar plant microbiomes

Emilia A. Mäkinen[1]*, Ole Franz[2], Janne A. Ihalainen[2], Marjo Helander[3], Riitta Nissinen[3], Suni Mathew[2,3], Irma Saloniemi[3], Kari Saikkonen[1]

[1]Biodiversity Unit, University of Turku, 20014 Turku, Finland

[2]Nanoscience Center and Department of Biological and Environmental Science, University of Jyväskylä, 40014 Jyväskylä, Finland

[3]Department of Biology, University of Turku, 20014 Turku, Finland

***Corresponding author**

Emilia A. Mäkinen

Address: Vesilinnantie 5, Natura building, Department of Biology, University of Turku, 20014 Turku, Finland

e-mail: emamak@utu.fi

ORCID: 0009-0002-5269-4262

Explorative colony analysis on the *Deschampsia antarctica* isolates

Statistical analysis

Results of the statistics conducted in R ver 4.3.2 (R Core Team 2023) were plotted using packages 'ggplot2', and 'ggpubr' (Wickham 2016; Kassambara 2023). To analyze differences in AAP activity between plants from Antarctica and Patagonia, the average proportion of AAP-positive colonies was compared between the origins using t-tests. Differences in total AAP proportions were compared by applying t-tests with unequal variances, followed by separate tests for the sampled plant parts (root endophytes, leaf endophytes, and phyllosphere) (Table S1). T-tests were conducted both using non-transformed data and arcsine-transformed proportions, but as the results of these tests did not differ, we report only the non-transformed.

The proportions of AAP-positive colonies from Antarctic and Patagonian samples included a high number of zeroes (no AAP activity), and the distribution of the data did not follow binomial or negative binomial distributions. As such, we chose to analyze differences in AAP activity via two approaches: qualitatively (AAPB presence/absence) with binomial models on a logit link, and quantitatively by excluding zeros (response as the total number of AAP-positive colonies) with Poisson models on a log link. These generalized linear mixed models were conducted separately for the sampled plant parts (root endophytes, leaf endophytes, and phyllosphere). Applying procedure glmer from package 'lme4' (Bates et al. 2015), origin (Antarctica or Patagonia), plant tissue (root endophytes, leaf endophytes, and phyllosphere), and interaction were used as fixed effects with sampling location as a random factor. Models with numerical latitude as the predictor instead of origin were also considered, and the results remained consistent. The fit of all models was assessed by running dispersion tests and interpreting simulated residual plots with package 'DHARMa' (Hartig 2022). The response variables of the models were tested using type II Wald's chi-square tests with package 'car' (Fox and Weisberg 2019). However, the significance of the results produced by the quantitative Poisson models is left uncertain, as model assumptions were not entirely fulfilled.

Results

At 6.3% and 6.7%, respectively, we found the mean proportion of AAP-positive colonies to remain similar ($t = -0.18$, $df = 106.2$, $p = 0.86$) between Antarctic and Patagonian plants (Fig S1; Table S1). For leaf endophytes, the mean proportion of AAP-positive colonies was higher ($t = -2.17$, $df = 33.2$, $p = 0.04$) in Patagonian plants (mean = 0.106, SE = 0.010) than in plants of Antarctic origin (mean = 0.047, SE = 0.010). Although the difference remained non-significant in root endophytes ($t = 1.00$, $df = 26.2$, $p = 0.33$) and the phyllosphere ($t = 0.59$, $df = 26.3$, $p = 0.56$), the mean proportion of AAP-positive colonies in root endophytes and the phyllosphere was higher in Antarctic plants (mean = 0.023, SE = 0.011; mean = 0.118, SE = 0.049) compared to Patagonian plants (mean = 0.010, SE = 0.005; mean = 0.086, SE = 0.022).

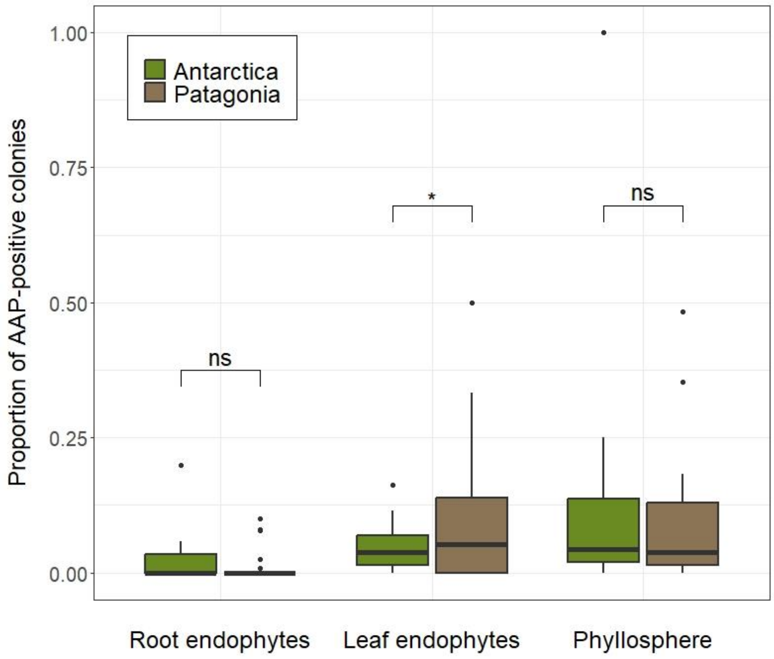

Fig S1. The mean proportion of AAP-positive colonies between Antarctica and Patagonia in different plant parts (root endophytes, leaf endophytes, and phyllosphere) is shown as boxplots (between 25th and 75th percentiles with medians as the horizontal lines together with 1.5 * interquartile whiskers and outliers shown as points). AAP activity in the plant parts was compared between the two locations with t-tests (Table S1), * $p < 0.05$, ns $p > 0.05$. Means are based on raw data.

Table S5. Results of t-tests on the proportions of AAP-positive colonies between Antarctica and Patagonia. T-tests were conducted separately for total proportions and proportions in sampled plant parts. SE = standard error.

				**Antarctica**	**Patagonia**
	df	t	p	mean ± SE	mean ± SE
**Total**	106.24	0.176	0.861	0.063 ± 0.018	0.067 ± 0.012
**Root endophytes**	26.21	1.002	0.326	0.023 ± 0.011	0.010 ± 0.005
**Leaf endophytes**	33.23	2.166	**0.038**	0.047 ± 0.010	0.106 ± 0.010
**Phyllosphere**	26.28	0.594	0.558	0.118 ± 0.049	0.086 ± 0.022

A quantitative approach to analyzing the strength of AAP activity corresponds with the results of the t-tests (Tables S1 and S2). This method compares the number of AAP-positive colonies to the total number of colonies in a sample, but only in samples where there was a positive AAP response. As such, observations

with zero AAPB colonies are not included in this analysis. Similar to the binomial approach (Fig 2), this comparison showed a significant divergence across plant samples in the intensity of AAP activity in different plant parts (χ^2 = 155.185, df = 2, p < 0.001). Though the difference between regions remained non-significant, a significant interaction was found between geographic region and AAP signal in different plant tissues (χ^2 = 92.997, df = 2, p < 0.001). Generally, endophytic AAPB were more abundant in the leaves of Patagonian plants (model estimate = 0.109, 95% CI = 0.061–0.193) compared to Antarctic plants (model estimate = 0.048, 95% CI = 0.024–0.097). This difference may be related to milder climate conditions and higher plant diversity found at lower latitudes (Harrison and Griffin 2020; Yang et al. 2023). In contrast, Antarctic plants had a higher AAP signal in the phyllosphere (model estimate = 0.090, 95% CI = 0.039–0.206) in comparison to Patagonian plants (model estimate = 0.059, 95% CI = 0.029–0.119). As plant surfaces provide a relatively hostile environment for microbe communities, differences in phyllospheric AAPB availability may be connected to prevailing climate conditions and plant-microbe interactions (Lindow and Brandl 2003; Vorholt 2012). On the other hand, the difference in root endophytes remained minor: model estimates 0.041 (95% CI = 0.024–0.069) and 0.039 (95% CI = 0.021–0.073) for Antarctic and Patagonian plants, respectively. A lower AAPB occurrence is expected from the root endosphere, as ambient light availability is low in below-ground plant parts. However, these findings may be biased by deviations in model residuals resulting from high within-population variability.

Table S6. Results of the statistical comparison of AAP activity between Antarctica and Patagonia. The analyses are based on two approaches applying generalized linear mixed models: a binomial response and a Poisson distributed response with zeroes excluded. The binomial model is based on a logit link, whereas the model with zeroes removed is applied on a log link. Df = degrees of freedom.

	**Binomial model**			**Poisson model**		
Fixed effect	df	Chisq	p	df	Chisq	p
Plant part	2	30.744	**<0.001**	2	155.185	**<0.001**
Region	1	1.676	0.196	1	0.174	0.676
Plant part x Region	2	0.875	0.646	2	92.997	**<0.001**

References

Bates D, Mächler M, Bolker B, Walker S (2015) Fitting Linear Mixed-Effects Models Using **lme4**. J Stat Softw 67:

Fox J, Weisberg S (2019) An R Companion to Applied Regression, 3rd Ed. Sage Publications, Thousand Oaks CA

Harrison JG, Griffin EA (2020) The diversity and distribution of endophytes across biomes, plant phylogeny and host tissues: how far have we come and where do we go from here? Environ Microbiol 22: 2107–2123

Hartig F (2022) DHARMa: Residual Diagnostics for Hierarchical (Multi-Level / Mixed) Regression Models

Kassambara A (2023) ggpubr: ‘ggplot2’ Based Publication Ready Plots

Lindow SE, Brandl MT (2003) Microbiology of the Phyllosphere. Appl Environ Microbiol 69:

R Core Team (2023) R: A Language and Environment for Statistical Computing

Vorholt JA (2012) Microbial life in the phyllosphere. Nat Rev Microbiol 10: 828–840

Wickham H (2016) ggplot2: Elegant Graphics for Data Analysis, 2nd Ed. Springer International Publishing, New York

Yang X, Wang P, Xiao B, Xu Q, Guo Q, Li S, Guo L, Deng M, Lu J, Liu L, Ma K, Schmid B, Jiang L (2023) Different assembly mechanisms of leaf epiphytic and endophytic bacterial communities underlie their higher diversity in more diverse forests. J Ecol 111: 970–981
